# Detecting and Measuring Depression on Social Media Using a Machine Learning Approach: Systematic Review

**DOI:** 10.2196/27244

**Published:** 2022-03-01

**Authors:** Danxia Liu, Xing Lin Feng, Farooq Ahmed, Muhammad Shahid, Jing Guo

**Affiliations:** 1 School of Sociology Huazhong University of Science and Technology Wuhan China; 2 Department of Health Policy and Management School of Public Health Peking University Beijing China; 3 Department of Anthropology University of Washington Seattle Seattle, WA United States; 4 Department of Anthropology Quaid-I-Azam University Islamabad Islamabad Pakistan; 5 School of Insurance and Economics University of International Business and Economics Beijing China

**Keywords:** depression, machine learning, social media

## Abstract

**Background:**

Detection of depression gained prominence soon after this troublesome disease emerged as a serious public health concern worldwide.

**Objective:**

This systematic review aims to summarize the findings of previous studies concerning applying machine learning (ML) methods to text data from social media to detect depressive symptoms and to suggest directions for future research in this area.

**Methods:**

A bibliographic search was conducted for the period of January 1990 to December 2020 in Google Scholar, PubMed, Medline, ERIC, PsycINFO, and BioMed. Two reviewers retrieved and independently assessed the 418 studies consisting of 322 articles identified through database searching and 96 articles identified through other sources; 17 of the studies met the criteria for inclusion.

**Results:**

Of the 17 studies, 10 had identified depression based on researcher-inferred mental status, 5 had identified it based on users’ own descriptions of their mental status, and 2 were identified based on community membership. The ML approaches of 13 of the 17 studies were supervised learning approaches, while 3 used unsupervised learning approaches; the remaining 1 study did not describe its ML approach. Challenges in areas such as sampling, optimization of approaches to prediction and their features, generalizability, privacy, and other ethical issues call for further research.

**Conclusions:**

ML approaches applied to text data from users on social media can work effectively in depression detection and could serve as complementary tools in public mental health practice.

## Introduction

Over recent decades, depression has increasingly become a matter of global public health concern [1]. The total number of people living with depression globally increased by 18.4% between 2005 and 2015. In 2015, more than 332 million (4.4%) people around the globe were found to be living with depression [2]. Mental disorders like depression rank 9th among global causes of disease burden, following common diseases such as stroke, heart diseases, and AIDS, and it can impair physical, as well as emotional, and mental health [3]. People with depression experience sleep disorders, lack of energy, low interest in daily activities, feelings of worthlessness, inability to concentrate, and recrudescent suicidality [4]. Detection of depression is critical for helping to relieve these threats.

Traditionally, depression is detected using standardized scales requiring patients’ subjective responses or clinical diagnoses given by attending clinicians—methods that have some shortcomings. Firstly, people’s responses to standardized scales administered in the traditional way are likely to be affected by context, the patient’s mental status at the time, the relationship between the clinician and the patient, the patient’s current mood, and the patient’s past experiences and memory bias. Traditional diagnostic methods also lack temporal granularity [5]. Secondly, people may be unaware or ashamed of their depressive symptoms and unlikely to consult with professional clinicians, especially in the early stages of depression. A previous study found that more than 70% of the population would not consult with professional clinicians if they were in the early stages of depression, meaning that they would be likely to allow their symptoms to worsen before they would consider seeking help [6]. Finally, detection of depression by traditional methods, being dependent on face-to-face interviews, is costly in terms of both money and time and unaffordable for some people [7]. Therefore, a more cost-effective method for detecting cases of depression, applicable to large populations, is needed.

Fortunately, application of the machine learning (ML) approach to text data from social media can provide an effective solution to this question. Social media such as Twitter, Facebook, discussion forums, and microblogs have long since become popular platforms for expressing and recording individuals’ personalities, feelings, moods, thoughts, and behaviors. Social media in this review refers to a cluster of applications that build upon technological and ideological foundations [8]. There were researchers classifying social media according to theories in the field of social processes consisting of self-presentation and self-disclosure. Self-presentation defines that people have the desire to get command of the impressions that other people have of them [9], and it is achieved through self-disclosure. Kaplan and Haenlein [8] classified social media relied on the type of self-presentation and the degree of self-disclosure. Different types of social media can help users conduct different types of self-presentation, such as text-based, video-based, picture-based, etc. And some groups of social media (eg, blogs and social networking sites) have a higher degree of self-disclosure. Hence, data mining of the vast quantities of text through which we can seek out the users found on social media can be of great value for detecting cases of depression [10]. In addition, ML, which has been developing rapidly in recent years, can help text mining and sentiment analysis to become more accurate and intelligent [11]. ML is a subfield of computer science that explores the construction and study of algorithms that can learn from and make predictions on data [12]. With recent and rapid advances in social media technology, mental health researchers have an opportunity to collect vast amounts of online data related to people’s mental states, and ML can serve as a robust technique for analyzing these data and detecting trajectories and dimensions of mental disorders (eg, depression and anxiety) [13]. For example, researchers in Australia proposed several ML models for predicting depressive symptoms among users based on text data from Reddit, and the models achieved high predictive precision. As a result, their ML approach was shown to potentially be a useful tool for monitoring social media user populations for early traces of depression and a complementary tool to well-established methods of depression detection [14]. In recent years, researchers have devoted considerable time and effort to developing ML approaches that can make use of words, topics, and other information contained in social media texts for detecting depression [14,15].

As far as we know, there are few existing reviews of ML approaches to depression detection that use text data from social media. Some previous reviews have focused on ML applications that use neuroimaging data to predict depression. For example, Mumtaz et al [16] conducted a detailed review of studies of the use of electroencephalogram and event-related potential data sets to detect major depressive disorder using ML approaches. Orrù et al [17] provided an overview of studies identifying imaging biomarkers of psychiatric diseases, such as major depression, using support vector machines (SVMs). There has also been a review focusing on studies about screening for mental illnesses by applying various methods to social media [18]. None of the existing reviews have focused on the application of ML approaches to texts from social media. However, ML approaches have unique advantages in the detection of depression using text data from social media. With people’s memberships in online forums, and their public sharing via the internet, text data from social media records are a treasure trove of psychological data, which can play a vital role in screening for depressive symptoms among users of social media. ML techniques also offer opportunities for identifying hidden patterns in online communication and interaction on social media that may reveal users’ mental states such as depression, anxiety, anger, etc [19]. Automatic detection of depressive symptoms through ML algorithms applied to social media data has potential as a way of identifying people at risk of depression through large-scale monitoring of online social networks and could complement traditional screening procedures. Systematic reviews of studies using ML approaches and text data from social media to detect depression can help provide directions for future research in the area, and help guide optimization of data mining, feature extraction, and processing methods so that the limitations of previous studies can be overcome, and prediction accuracy and generalization capability improved. Such reviews should describe the depression identification and classification methods being used.

In this paper, we systematically reviewed studies that adopted the ML approach to measure depressive symptoms based on any text mining techniques to identify sentiments using social media data. We specified the ML methods that were used to identify mental status and discuss the evolution of the methods and their pros and cons and provide suggestions for future research in the area.

## Methods

### Search Strategy

**Figure 1:**
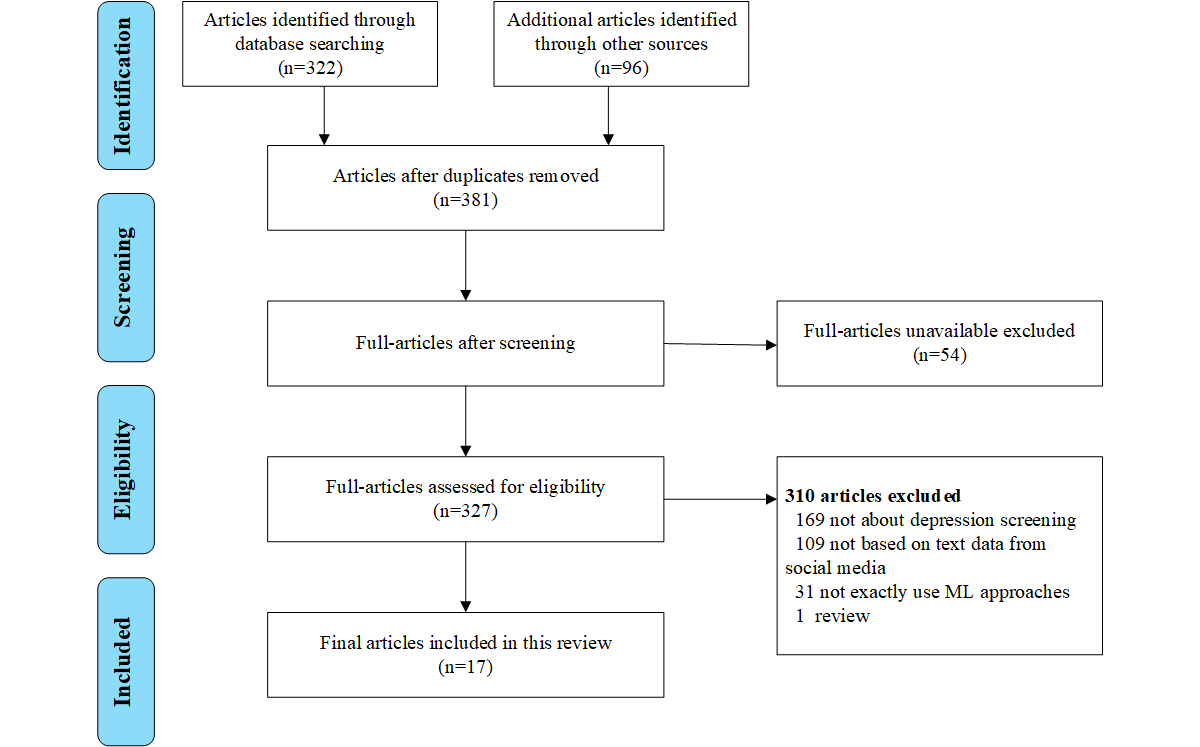
Flowchart for the systematic search of studies in this review.

We searched several English- and Chinese-language online bibliographic databases for relevant articles, specifically, Google Scholar, PubMed, Medline, ERIC, PsycINFO, and BioMed, and the Chinese Wangfang, Weipu, and China National Knowledge Infrastructure databases. Our search placed no restrictions on publication type. However, because the age of social media began in the 1990s [8], we did restrict the papers’ publication dates to the period between January 1990 and December 2020. Search strings related to ML, depression detection, social media, and text were utilized, that is “Machine Learning” or “Deep Learning” or “Artificial Intelligence” AND “Depression detection” or “screening depression” or “predicting depression” or “recognizing depression” or “major depressive disorder” AND “social media” or “social network” or “online” or “Twitter” or “micro-blog” or “web post” or “Facebook” or “Reddit” or “LiveJournal” or “WeChat” AND “text.” We aimed to find studies focusing on the use of ML approaches, such as SVMs, Bayes, latent Dirichlet allocation (LDA), decision tree, and neural networks to detect depression through text mining from social media. “Text mining” refers to mining online textual posts of social media users, including those containing emoticons. And it is worth mentioning that we collect the articles that only screen depression, not those studying several symptoms, which include depression. For example, we first input “machine learning,” “depression screening,” and “social media” in Google Scholar and obtained more than 20000 articles published during the period between January 1990 and December 2020. We made a preliminary judgment based on the title and abstract to identify the studies we needed. Most of the 20,000 articles do not meet the criterion. Some of the articles do not use text data from social media, but videos, photos, etc. There are also some articles that are depression-related, but they do not detect, screen, or predict depression. This search retrieved 322 articles, all dealing with depression, social media, and ML. We also collected 96 relevant articles that were cited in the 322 articles thus retrieved. After the reviewers screened the retrieved citations according to a set of exclusion criteria, seventeen of them were selected for inclusion in this review. Figure 1 shows the process by which the final set of seventeen studies was selected.

### Study Selection

The article titles and abstracts were screened independently by 2 reviewers (JG and DL). The reviewers then retrieved and assessed the available full texts of the studies and excluded articles that (1) did not discuss ML approaches or detection of depression, (2) were not focused on the use of textual (as opposed to video and image) data from social media, or (3) were themselves reviews of existing research on the use of texts from social media to detect depressive symptoms with ML approaches. The 2 reviewers also recorded important data about the articles such as authors, sample size, platform, study design, assessment tools, outcome of interest, and findings. Disagreements concerning particular articles were resolved through discussions aimed at reaching consensus. Details of the process are shown in Figure 1.

### Quality Assessment

The study quality assessment for the 17 studies included was conducted by 2 independent reviewers, using the 14-item NIH Quality Assessment Tool for Observational Cohort and Cross-Sectional Studies [20]. For each study, they gave each of the checklist items a score of 0 (no) or 1 (yes). The total scores ranged from 0 to 14. Therefore, each reviewer classified each study as low (6), medium (7-10), or high (14) quality and then assigned a quality score to each one. Any discrepancies between the 2 reviewers’ ratings were discussed, and a consensus rating was recorded. These consensus ratings are shown in Table 1.

**Table 1:** Study quality assessment.

Reference	Q1^a^	Q2^b^	Q3^c^	Q4^d^	Q5^e^	Q6^f^	Q7^g^	Q8^h^	Q9^i^	Q10^j^	Q11^k^	Q12^l^	Q13^m^	Q14^n^	Total Score	Rank
Wang et al [21], 2013	1	1	1	1	1	0	1	1	1	1	1	0	1	1	12	high
Burdisso et al [14], 2019	1	1	1	1	1	0	1	1	1	1	1	0	1	1	12	high
Nguyen et al [22], 2014	1	0	0	1	1	0	1	1	1	1	1	0	1	1	10	medium
Fatima et al [23], 2018	1	0	0	1	1	0	1	1	1	1	1	0	1	1	10	medium
Tung & Lu [15], 2016	0	0	1	1	1	0	1	1	1	1	1	0	0	1	9	medium
Husseini Orabi et al [24], 2018	1	0	1	1	1	0	1	1	1	1	1	0	0	1	10	medium
Islam et al [19], 2018	1	1	0	1	1	0	1	1	1	1	1	1	0	1	11	high
Shen et al [6], 2017	0	1	0	1	0	0	1	1	1	1	1	1	0	1	9	medium
De Choudhury, Gamon [25], et al, 2013	0	0	1	1	1	0	1	1	1	1	1	0	1	1	10	medium
Mariñelarena-dondena et al [26], 2017	0	1	1	1	1	0	1	1	1	1	1	0	0	1	10	medium
Tsugawa et al [27], 2015	1	1	0	1	1	1	1	1	1	1	1	1	1	1	13	high
Chen et al [28], 2018	0	0	0	0	0	0	1	1	1	1	1	0	0	1	6	low
De Choudhury, Counts [29], et al, 2013	0	0	0	1	1	0	1	1	1	1	1	1	0	1	9	medium
Dinkel et al [30], 2019	0	0	0	0	0	0	1	1	1	1	1	0	0	1	6	low
Sadeque et al [7], 2017	1	1	1	1	1	0	1	1	1	1	1	1	0	1	12	high
Shatte et al [31], 2020	1	1	1	1	1	1	1	0	1	1	1	0	1	1	12	high
Li et al [32], 2020	1	1	1	1	0	1	1	0	1	1	1	0	1	0	10	medium

^a^Q1: Was the research question or objective in this paper clearly stated?

^b^Q2: Was the study population clearly specified and defined?

^c^Q3: Was the participation rate of eligible persons at least 50%?

^d^Q4: Were all the subjects selected or recruited from the same or similar populations? Were inclusion and exclusion criteria for being in the study prespecified and applied uniformly to all participants?

^e^Q5: Was a sample size justification, power description, or variance and effect estimates provided?

^f^Q6: Were the exposure(s) of interest measured before the outcome(s) were measured?

^g^Q7: Was the timeframe sufficient so that one could reasonably expect to see an association between exposure and outcome if it existed?

^h^Q8: For exposures that can vary in amount or level, did the study examine different levels of the exposure in relation to the outcome?

^i^Q9: Were the exposure measures clearly defined, valid, reliable, and implemented consistently across all study participants?

^j^Q10: Were the exposure(s) assessed more than once over time?

^k^Q11: Were the outcome measures clearly defined, valid, reliable, and implemented consistently across all study participants?

^l^Q12: Were the outcome assessors blinded to the exposure status of participants?

^m^Q13: Was loss to follow-up after baseline 20% or less?

^n^Q14: Were key potential confounding variables measured and their impact on the relationship between exposure(s) and outcome(s) statistically adjusted for?

## Results

### Depression Identification

The samples, methods, and results of the 17 studies that met the inclusion criteria are summarized in Table 2. This review will further summarize the depression identification and ML methods used in the 17 studies (Table 3). Nine of the studies [15,21,25,27-32] identified depression based on researcher-inferred mental status, while 6 [6,7,14,19,24,26] used user-declared mental status, and 2 [22,23] identified it based on community membership.

**Table 2:** Summary of machine learning studies of detection of depression using text data from social media.

Reference	Sample	Platform	Outcome	Depression identification method	ML^a^ approach type	Features examined	Cross- validation	Type of study
Wang et al [21], 2013	122 depressed and 346 nondepressed subjects, the ages of the samples were not reported	Sina microblog	Bayes: mean absolute error=0.186, ROC^b^=0.908, F-measure=0.85; Trees: mean absolute error=0.239, ROC=0.798, F-measure=0.762; Rules: mean absolute error=0.269, ROC=0.869, F-measure=0.812	Researcher-inferred	3 classification approaches: Bayes, trees and rules	Ten features from three dimensions, including microblog content, interactions, and behaviors. Four of the ten features, (1st person singular, 1st person plural, positive emoticons, and negative emoticons) pertain to microblog content, while three pertain to interactions (mentioning, [being] forwarding, and commenting), and two pertain to behaviors (original blogs and blogs posted between midnight and 6:00 am).	10-fold cross-validation	Observational cohort study
Burdisso et al [14], 2019	486 training subjects (83 depressed/403 nondepressed); 401 test subjects (52 depressed/349 nondepressed), the ages of the samples were not reported	Reddit	SS3^c^: F-measure=0.61, precision=0.63, recall=0.60	User-declared	The proposed model: SS3	Words in users’ online text posts on Reddit	4-fold cross-validation	Observational cohort study
Nguyen et al [22], 2014	5000 posts made by users from clinical communities and 5000 posts from control communities, the ages of the samples were not reported	LiveJournal	Lasso to classify communities (Accuracy): ANEW^d^=0.89, mood=0.96, topic=1, LIWC^e^=1; Lasso to classify posts (Accuracy): topic=0.93, LIWC=0.88	Community membership-based	The Lasso model	Affective features, mood tags, features topics from the LIWC, all extracted from posts on LiveJournal.	10-fold cross-validation	Observational cohort study
Fatima et al [23], 2018	4026 posts (2019/2007) from depressive and non-depressive communities, the ages of the samples were not reported	LiveJournal	The proposed RF^f^-based model (Accuracy): post=0.898, community=0.950, depression degree=0.923; SVM^g^ (Accuracy): post=0.8, community=0.895	Community membership-based	Random forest, SVM	The values of the feature set serve as inputs to the classification algorithm, being extracted from first person singular, positive emotion, negative emotion, anxiety, cognitive process, insight, cause, affiliation health, and informal language of online text.	10-fold cross-validation	Observational cohort study
Tung & Lu [15], 2016	724 posts, the ages of the samples were not reported	PTT^h^	EDDTW^i^: precision=0.593, recall=0.668, F-measure=0.624	Researcher-inferred	EDDTW	Negative emotion lexicon, negative thought lexicon, negative event lexicon, and symptom lexicon.	10-fold cross-validation	Observational cohort study
Husseini Orabi et al [24], 2018	154 subjects (53 labeled as Depressed/101 labeled as Control), the ages of the samples were not reported	Twitter	The optimized CNN^j^ model: accuracy=0.880	User-declared	CNN-based models, RNN^k^-based models, SVM	Twitter texts from among which all the @mentions, retweets, nonalphanumeric characters, and URLs were extracted by the researchers.	5-fold cross-validation	Observational cohort study
Islam et al [19], 2018	7145 Facebook comments (58% depressed/42% nondepressed), the ages of the samples were not reported	Facebook	Decision Tree (F-measure): emotional process=0.73, linguistic style=0.73, temporal process=0.73, all features=0.73; SVM (F-measure): emotional process=0.73, linguistic style=0.73, temporal process=0.73, all features=0.73; KNN^l^ (F-measure): emotional process=0.71, linguistic style=0.70, temporal process=0.70, all features=0.67; Ensemble (F-measure): emotional process=0.73, linguistic style=0.73, temporal process=0.73, all features=0.73	User-declared	SVM, decision tree, ensemble, KNN	Emotional information (positive, negative, anxiety, anger, and sad), linguistic style (prepositions, articles, personal, conjunctions, auxiliary verbs), temporal process information (past, present, and future)	10-fold cross-validation	Observational cohort study
Shen et al [6], 2017	1402 depressed users, 36993 depression-candidate users, and over 300 million nondepressed users, the ages of the samples were not reported	Twitter	Accuracy: NB^m^=0.73, MSNL^n^=0.83, WDL^o^=0.77, MDL^p^=0.85	User-declared	MDL, NB, MSNL, WDL	Features of network interactions (number of tweets, social interactions, and posting behaviors), user profiles (users’ personal information in social networks), and visual, emotional, and topic-level features, domain-specific features	5-fold cross-validation	Observational cohort study
De Choudhury, Gamon, et al [25], 2013	476 users (171 depressed/305 nondepressed), with a median age of 25	Twitter	Accuracy: engagement=0.553, ego-network=0.612, emotion=0.643, linguistic style=0.684, depression language=0.692, demographics=0.513, all features=0.712	Researcher-inferred	SVM	Engagement, egocentric social graph, emotion, linguistic style, depression language, demographics	10-fold cross-validation	Observational cohort study
Mariñelarena-dondena et al [26], 2017	135 articles (20 depressed/115 nondepressed), the ages of the samples were not reported	Reddit	Precision=0.850, recall=0.810, F-measure=0.829, accuracy=0.948	User-declared	SVD, GBM^q^, SMOTE^r^	n-grams, use of which can create a large feature space and hold much important information	Not reported	Observational cohort study
Tsugawa et al [27], 2015	209 Japanese users (81 depressed/128 nondepressed), and users were aged 16-55, with a median age of 28.8 years	Twitter	Precision=0.61, recall=0.37, F-measure=0.46, accuracy=0.66	Researcher-inferred	LDA^s^, SVM	Frequencies of words used in the tweet, ratio of tweet topics found by LDA, ratio of positive-affect words contained in the tweet, ratio of negative-affect words contained in the tweet, hourly posting frequency, tweets per day, average number of words per tweet, overall retweet rate, overall mention rate, ratio of tweets containing a URL, number of users following, number of users followed	10-fold cross-validation	Observational cohort study
Chen et al [28], 2018	446 perinatal users, the ages of the samples were not reported	WeChat circle of friends	The result of LSTM^w^ was similar to EPDS^x^	Researcher-inferred	LSTM	Top 10 emotions in the data set	Not reported	Observational cohort study
De Choudhury, Counts, et al [29], 2013	489 users, with a median age of 25 years	Twitter	Accuracy: eng.+ego=0.593, n-grams=0.600, style=0.658, emo.+ time=0.686, all features=0.701	Researcher-inferred	PCA^t^, SVM	Postcentric features (emotion, time, linguistic style, n-grams), user-centric features (engagement, ego-network)	5-fold cross-validation	Observational cohort study
Dinkel et al [30], 2019	142 speakers (42 depressed/100 nondepressed), the ages of the samples were not reported	Distress Analysis Interview Corpus-Wizard of Oz (WOZ-DAIC) database	Precision=0.93, recall=0.83, F-measure=0.87	Researcher-inferred	LSTM	Words from online posts	10-fold cross-validation	Observational cohort study
Sadeque et al [7], 2017	888 users (136 depressed/752 nondepressed), the ages of the samples were not reported	Reddit	F-measure: LibSVM^u^=0.40, WekaSVM^v^=0.30, RNN=0.34, Ensemble=0.45	User-declared	LibSVM, RNN, Ensemble, WekaSVM	Depression lexicon^y^, metamap features^z^	5-fold cross-validation	Observational cohort study
Shatte et al [31], 2020	365 fathers in the perinatal period, the ages of the samples were not reported	Reddit	Precision=0.67, recall=0.68, F-measure=0.67, accuracy=0.66	Researcher-inferred	SVM	Fathers’ behaviors, emotions, linguistic style, and discussion topics	10-fold cross-validation	Observational cohort study
Li et al [32], 2020	1,410,651 users, the ages of the samples were not reported	Twitter	Accuracy:SVM (radial basis function kernel)=0.82, SVM (linear kernel)=0.87, logistic regression=0.86, naïve Bayes=0.81, simple neural network=0.87	Researcher-inferred	SVM, logistic regression, naïve Bayes Classifier, simple neural network	512 features that were extracted from tweets using a universal sentence encoder	Not reported	Observational cohort study

^a^ML: machine learning.

^b^ROC: receiver operating characteristic.

^c^SS3: sequential S3 (smoothness, significance, and sanction).

^d^ANEW: affective norms for English words.

^e^LIWC: linguistic inquiry and word count.

^f^RF: random forest.

^g^SVM: support vector machine.

^h^PTT: the gossip forum on the Professional Technology Temple.

^i^EDDTW: event-driven depression tendency warning.

^j^CNN: convolutional neural networks.

^k^RNN: recurrent neural network.

^l^KNN: k-nearest neighbor.

^m^NB: naive Bayesian.

^n^MSNL: multiple social networking learning.

^o^WDL: Wasserstein Dictionary Learning.

^p^MDL: multimodal depressive dictionary learning.

^q^GBM: gradient boosting machine.

^r^SMOTE: synthetic minority oversampling technique.

^s^LDA: latent Dirichlet allocation.

^t^PCA: principal component analysis.

^u^LibSVM: library for support vector machines.

^v^WekaSVM: Waikato Environment for Knowledge Analysis for support vector machines.

^w^LSTM: long short-term memory.

^x^EPDS: Edinburgh Postnatal Depression Scale.

^y^A cluster of unigrams that has a great likelihood of appearing in depression-related posts.

^z^The features were extracted using Metamap based on concepts from the Unified Medical Language System Metathesaurus.

**Table 3:** Summary of the studies’ depression identification methods.

Type of depression identification method and reference		Platform		Specific diagnostic method
**Researcher-inferred mental status**				
	De Choudhury, Gamon, et al [25], 2013	Twitter	CES-D^a^ questionnaire	
	De Choudhury, Counts, et al [29], 2013	Twitter	CES-D^a^ questionnaire	
	Tsugawa et al [27], 2015	Twitter	CES-D^a^ questionnaire	
	Chen et al [28], 2018	WeChat	The Edinburgh Postnatal Depression Scale (EPDS) questionnaire	
	Dinkel et al [30], 2019		The Patient Health Questionnaire (PHQ-8)	
	Li et al [32], 2020	Twitter	The Patient Health Questionnaire (PHQ)	
	Wang et al [21], 2013	Sina Microblog	Diagnosis by psychologists using interviews and questionnaires	
	Tung et al [15], 2016	PTT^b^	Diagnosis by three professional students	
	Shatte et al [31], 2020	Reddit	ICD-10^c^ and diagnosis by a clinical psychologist specializing in perinatal mental health	
**User-declared mental status**				
	Burdisso et al [14], 2019	Reddit	Statements specifically indicating depression, such as “I was diagnosed with depression.”	
	Mariñelarena-dondena et al [26], 2017	Reddit	Documents declaring depression diagnoses	
	Sadeque et al [7], 2017	Reddit	Statements like “I have been diagnosed with depression.”	
	Husseini Orabi et al [24], 2018	Twitter	Documents declaring depression diagnoses	
	Shen et al [6], 2017	Twitter	Tweets of statements like “I was diagnosed with depression.”	
	Islam et al [19], 2018	Facebook	Indication of depression by ground truth label information on selected posts	
**Community membership**				
	Nguyen et al [22], 2014	LiveJournal	Five “clinical” communities and five “control” communities	
	Fatima et al [23], 2018	LiveJournal	Five depressed and five nondepressed communities	

^a^CES-D: Center For Epidemiologic Studies Depression Scale.

^b^PTT: the gossip forum on the professional technology temple.

^c^ICD-10: International Classification Of Diseases, tenth revision.

### Identification Based on Researcher-Inferred Mental Status

Researcher-inferred mental status means that researchers identified the users’ mental status based on the content of the users’ online posts using ML approaches and professional diagnostic scales or expert opinions. Among the studies reviewed, 9 out of 17 studies [15,21,25,27-32] identified depression based on researcher-inferred mental status, while 6 [25,27-30,32] used professional diagnostic scales, and in 2 studies [15,21], depressive tendencies were identified in a traditional way, by clinical professionals; 1 study [31] used both diagnostic scales and expert opinions.

In particular, De Choudhury et al [25] conducted 2 studies using text data from Twitter in 2013. The first study collected data on 476 subjects who had reported depressive symptoms during September 2011-June 2012—among them 171 depressed users and 305 nondepressed users. The second study included 251 male and 238 female users, whose median age was 25 years [29].

In addition, Tsugawa et al [27] implemented data gathered from Japanese-speaking users through the Twitter application programming interface (API). They collected data on 209 participants—among them 121 males and 88 females aged 16 to 55 years, from December 4, 2013, to February 8, 2014, with a depression incidence of about 39%. The authors discuss the fact that Japanese personal pronouns work quite differently from those in Western languages, and subject words are often absent in Japanese texts, which could influence the performance of models being applied across different language contexts. And then, in a Chinese study, Chen et al [28] employed emoticon data from a WeChat circle of friends to detect perinatal depression. They gathered data on 446 perinatal participants, who had posted 1.17 million texts on the WeChat platform, 80% of the group being used as the training set, and the other 20% as the test set.

Finally, Dinkel et al [30] acquired text data from the Distress Analysis Interview Corpus-Wizard of Oz (WOZ-DAIC), a database that is publicly available. They combined data from 107 interviewees from the training set and 35 from the development set. The interviewees from the training set had a depression incidence of 28%, while those in the development set had an incidence of 34%.

In addition, Li et al [32] conducted a study using an ML approach to detect depression during the early COVID-19 outbreaks in the United States, based on the researcher-inferred depressive symptoms on Twitter. They collected tweet posts from 1,410,651 users, which were over 0.4% of the total population.

One of the 2 studies in which depression was identified in a traditional way was Wang et al’s [21]. They collected data on users of Sina microblog, which is one of the most popular social network services in China. They collected information from 6013 microblogs dating from August 1-15, 2012, and thus identified 122 depressed and 346 nondepressed subjects from among several hundred who had volunteered for the study. The second study, Tung et al [15], gathered about 18,000 web posts from the Chinese-language online forum PTT (the Gossip Forum on the Professional Technology Temple) from March 2004 to September 2011, of which 724 posts were selected as testing and training data. Next, a Chinese word segmentation and part-of-speech labeling tool was used for sorting and labeling the posts.

One other study combined depression diagnostic criteria with expert opinions. Shatte et al [31] studied the depression-related changes in mood among fathers who reported the births of children on Reddit posts. The study collected social media data on the fathers during the prepartum and postpartum periods and assessed features including behaviors, emotions, linguistic styles, and discussion topics, as well as more basic information.

### Identification Based on User-Declared Mental Status

“User-declared mental status” means that users declared, in social media posts, that they had been diagnosed with depression. Six studies used depression identification based on user declarations of mental status in the social media data. Burdisso et al [14] divided the data gathered from users on Reddit into a training set and a test set. The last data set included 135 depressed users and 752 nondepressed ones. Mariñelarena-dondena et al [26] constructed a data set containing 486 submissions, including posts and comments gathered from members of the Reddit community between February 2017 and April 2017. The final data set consisted of 83 depressed users and 403 nondepressed ones. And Sadeque et al [7] used the Reddit API to conduct data collection and constructed a data set of posts by 888 Redditors, among whom 136 were depressed, and 752 were nondepressed.

Two other studies constructed their experimental data sets using Twitter data. Husseini Orabi et al [24] selected 154 users whose Twitter labels were depressed or nondepressed. They also used the users’ posts published under the Bell Let's Talk campaign, and the final data collection consisted of 53 depressed users and 101 nondepressed. The second study, Shen et al [6], gathered data from users whose tweets had stated “I was diagnosed with depression” on Twitter. Altogether, they collected 292,564 tweets that had been posted by 1402 depressed subjects over the course of a month.

Islam et al [19] collected text data from Facebook in order to explore ways of detecting depression. Of the total of 7145 posts that they collected, 58% were from depressed users, and 42% were from nondepressed ones.

### Identification Based on Community Membership

Two studies that both explored depression identification, using community membership as an identifier, collected their data from LiveJournal. To construct a balanced data set, Nguyen et al [22] selected 5000 posts from five depressed (“clinical”) communities and 5000 posts from five nondepressed (“control”) communities. Fatima et al [23] also used data from five depressed and five nondepressed communities. Their final data set consisted of a total of 4026 posts, which included 2007 from nondepressed, and 2019 from depressed communities.

### The ML Approaches for Depression Detection

The ML approaches used in these studies included supervised learning (SL) and unsupervised learning (UL) approaches. SL methods specify a targeted outcome variable, such as the presence of a mental disorder, and are often used in prediction tasks. UL methods are used to detect relationships among the variables in a data set in the absence of a specified target outcome or response variable to supervise the analyses. UL aims to discover underlying structures such as clusters, components, or dimensions, in the data set [12]. Among the 17 ML-based studies reviewed here, 14 used SL approaches to explore depression detection methods, [7,14,15,19,21-25,27,28,30-32] and 3 used UL approaches [6,26,29] (Table 4).

**Table 4:** Summary of the machine learning approaches used in the depression detection studies.

Study			Machine learning approaches		Features		Outcomes
**Supervised learning approaches**							
	Nguyen et al [22], 2014	The Lasso model		Affective features, mood tags, thee linguistic inquiry and word count (LIWC) features and topics that were all extracted from posts on LiveJournal.		Community classification of user (Accuracy): ANEW=0.89, mood=0.96, topic=1, LIWC=1; Community classification of post (Accuracy): topic=0.93, LIWC=0.88	
	Chen et al [28], 2018	LSTM^a^		Top 10 emotions in the data set		Depression, according to the LSTM, and according to the EPDS^b^. The results were similar for both	
	Dinkel et al [30], 2019	LSTM		Words from online posts		Precision=0.93, recall=0.83, F-measure=0.87	
	Wang et al [21], 2013	Bayes,Trees, and Rules		Micro-blog content, interactions, and behaviors		Bayes: Mean absolute error=0.186, ROC=0.908, F-measure=0.85; Trees: Mean absolute error=0.239, ROC=0.798, F-measure=0.762; Rules: Mean absolute error=0.269, ROC=0.869,F-measure=0.812	
	Burdisso et al [14], 2019	The proposed model: SS3		Words in online text users posts on Reddit		SS3: F-measure =0.61, precision=0.63, recall=0.60	
	De Choudhury, Gamon et al [25], 2013	SVM^c^		Engagement, egocentric social graph, emotion, linguistic style, depression language, demographics		Accuracy: engagement=0.553, ego-network=0.612, emotion=0.643, linguistic style=0.684, depression language=0.692, demographics=0.513, all features=0.712	
	Tsugawa et al [27], 2015	LDA^d^, SVM		Frequencies of words used in the tweet, ratio of tweet topics found by LDA, ratio of positive-affect words contained in the tweet, ratio of negative-affect words contained in the tweet, hourly posting frequency, tweets per day, average number of words per tweet, overall retweet rate, overall mention rate, ratio of tweets containing a URL, number of users following, number of users followed		Precision=0.61, recall=0.37, F-measure=0.46, accuracy=0.66	
	Islam et al [19], 2018	SVM, decision tree, ensemble, KNN^e^		Emotional information (positive, negative, anxiety, anger, and sad), linguistic style (prepositions, articles, personal, conjunctions, auxiliary verbs), temporal process information (past, present, and future)		Decision Tree (F-measure): emotional process=0.73, linguistic style=0.73, temporal process=0.73, all features=0.73; SVM (F-measure): emotional process=0.73, linguistic style=0.73, temporal process=0.73, all features=0.73; KNN (F-measure): emotional process=0.71, linguistic style=0.70, temporal process=0.70, all features=0.67; Ensemble (F-measure): emotional process=0.73, linguistic style=0.73, temporal process=0.73, all features=0.73	
	Fatima et al [23], 2018	Random forests, SVM		The feature set values serve as an input to the classification algorithm, which were extracted from first person singular, positive emotion, negative emotion, anxiety, cognitive process, insight, cause, affiliation health, and informal language of text online.		The proposed RF-based model (Accuracy): post=0.898, community=0.950, depression degree=0.923; SVM (Accuracy): post=0.82, community=0.895	
	Shatte et al [31], 2020	SVM		Fathers’ behaviors, emotions, linguistic style, and discussion topics		Precision=0.67, recall=0.68, F-measure=0.67, accuracy=0.66	
	Husseini Orabi et al [24], 2018	CNN^f^-based models、RNN^g^-based models、SVM		Twitter text, among which all the @mentions, retweets, nonalphanumeric characters and, URLs were removed by researchers.		The optimized CNN model: accuracy=0.880	
	Sadeque et al [7], 2017	LibSVM, RNN, Ensemble, WekaSVM		Depression lexicon, metamap features		F-measure: LibSVM=0.40, WekaSVM=0.30, RNN=0.34, Ensemble=0.45	
	Li et al [32], 2020	SVM, logistic regression, naïve Bayes classifier, simple neural network		512 features that were extracted from tweets using a universal sentence encoder		Accuracy: SVM (radial basis function kernel)=0.82, SVM (linear kernel)=0.87, logistic regression=0.86, naïve Bayes=0.81, simple neural network=0.87	
	Tung et al [15], 2016	EDDTW^j^		Negative emotion lexicon, negative thought lexicon, negative event lexicon, and symptoms lexicon		Precision=0.593 Recall=0.668 F-measure=0.624	
**Unsupervised learning approaches**							
	Shen et al [6], 2017	MDL^k^, NB^l^, MSNL^m^, WDL^n^		Social network feature (number of tweets, social interactions, and posting behaviors), user profile feature (users’ personal information in social networks), visual feature, emotional feature, topic-level feature、domain-specific feature		Accuracy: NB=0.73, MSNL=0.83, WDL=0.77, MDL=0.85	
	De Choudhury, Counts et al [29], 2013	PCA^o^, SVM		Post-centric features (emotion, time, linguistic style, n-grams), user-centric features (engagement, ego-network)		Accuracy: eng.+ego=0.593, n-grams=0.600, style=0.658, emo.+time=0.686, all features=0.701	
	Mariñelarena-dondena et al [26], 2017	SVD^p^, GBM^q^, SMOTE^r^		n-grams that could produce large feature space and hold important information		Precision=0.850, recall=0.810, F-measure=0.829, accuracy=0.948	

^a^LSTM: long short-term memory.

^b^EPDS: Edinburgh Postnatal Depression Scale Questionnaire.

^c^SVM: support vector machine.

^d^LDA: latent Dirichlet allocation.

^e^KNN: k-nearest neighbor.

^f^CNN: convolutional neural networks.

^g^RNN: recurrent neural network.

^h^LibSVM: a library for support vector machines.

^i^WekaSVM: Waikato Environment for Knowledge for support vector machines.

^j^EDDTW: event-driven depression tendency warning.

^k^MDL: multimodal depressive dictionary learning.

^l^NB: naive Bayesian.

^m^MSNL: multiple social networking learning.

^n^WDL: Wasserstein Dictionary Learning.

^o^PCA: principal component analysis.

^p^SVD: singular value decomposition.

^q^GBM: gradient boosting machine.

^r^SMOTE: synthetic minority oversampling technique.

### Detection With Supervised Learning Approaches

The SL approaches used include regression and classification. Among the 14 studies that employed SL, 3 used regression [22,28,30], 8 adopted classification, [14,15,19,21,23,25,27,31], and 3 combined the two approaches [7,24,32].

Of the 3 that used regression-type SL approaches, Nguyen et al [22] found that the model performed best at community classification (accuracy of 100%) when linguistic inquiry and word count (LIWC) software and topics features were input, while affective feature and mood tags produced accuracies of 89% and 96%, respectively. Moreover, when LIWC and topics were used as feature sets in blog postclassification, performance was effective, with accuracies of 88% and 93%, respectively. Chen et al [28] conducted perinatal depression screening based on data from the WeChat circle of friends with a long short term memory (LSTM) network model. Their results indicated that the prediction power of LSTM was similar to that of the Edinburgh Postnatal Depression Scale, which has been demonstrated to be effective for perinatal depression detection. Similar to Chen et al’s study [28], Dinkel et al [30] also adopted LSTM to conduct depression detection based on data from the WOZ-DAIC database. They found that the behavioral aspects of texts were more useful for depression detection than the actual text content, and the proposed bidirectional long short-term memory model obtained the best performance, with the highest F1 score (0.87).

Additionally, 8 studies focused on depression detection using classification-type SL approaches [14,15,19,21,23,25,27,31]. Among the 8 studies, 4 focused on social media users [14,21,25,27], while 4 focused on submissions (comments and posts) [15,19,23,31]. Among the 4 studies focusing on users, Wang et al [21] focused on 468 users of the Sina Microblog, employing three types of approaches: rules, trees, and Bayes, all of which achieved accuracies around 80%. They discovered that users’ number of times of mentioning others was highly predictive of depression. Meanwhile, Burdisso et al [14] proposed the sequential S3 (smoothness, significance, and sanction; SS3) model to conduct depression screening using text data from 887 selected Reddit users, acquiring higher prediction accuracy than other models. The SS3 model would prompt the “large-scale passive monitoring” to be conducted online incrementally. It was not aimed at autonomous diagnosis but intended as a complementary tool to other more well-established methods for diagnosing psychological problems. They also stated that a set of legal and ethical questions related to data ownership and protection were open to debate. In addition, De Choudhury, Gamon et al [25] and Tsugawa et al [27] both used SVM approaches. De Choudhury, Gamon et al [25] conducted depression detection using SVM and obtained good performance, with an accuracy of 70%. Analyzing the behaviors of depressed users, they concluded that depressed users showed decreasing social activity, higher self-attentional focus, more negative emotion, increased expression of religious thoughts, and heightened medicinal and relational concerns. Among those not using SVM, Tsugawa et al [27] applied LDA, which performed with an accuracy of 66%. They found that the research results on effective depression predictors for Japanese users were different from those that were effective for people posting in English. Specifically, the number of times posting and mentioning others had good predictive power for English-based studies [21,25] but were not robust features in the Japanese study.

Of the 4 studies that focused on posting submissions, Tung et al [15] proposed an event-driven depressive tendency warning (EDDTW) model for detecting depressive tendencies based on posts on PPT networks, which showed the highest F-measure score for 0.624 of the EDDTW model, suggesting that EDDTW could be used to track trends or changes in depression among post authors. Fatima et al [23] used random forests and SVM to conduct classifications, achieving post and community classifications based on random forests with accuracies of 90% and 95%, and post and community classifications based on SVM with accuracies of 82% and 90%. Shatte et al [31] collected 3889 submissions and 63,907 comments from fathers reporting birth events over a 6-month period and assessed the data using linear support vector classification. They found that SVM with linear kernel produced the best prediction results. Besides, Islam et al [19] conducted depression prediction based on text data from Facebook, showing that decision trees acquired the highest accuracy in different experiments than other ML approaches.

Finally, 3 studies combined regression and classification [7,24,32]. Husseini Orabi et al [24] used convolutional neural networks (CNN), recurrent neural network (RNN), and SVM to predict depression, obtaining high accuracy of 88% with an optimized CNN model. The experiment indicated that CNN-based models performed better than RNN-based models for depression detection, and user-level classification could perform well in imbalanced or small data sets. Sadeque et al [7] predicted depression with a library for support vector machines, RNN, WekaSVM (Waikato Environment for Knowledge for support vector machines), and Ensemble models. They found the ensemble models performed better than the individual model and more data could improve traditional performance measures. Moreover, Li et al [32] proposed the correlation explanation (CorExQ9) algorithm that integrates with clinical stress measure index (PHQ-9) for depression detection using biweekly COVID-19 related language data from Twitter. And the innovative algorithm predicts depressive symptoms effectively and could be applied to other cases for stress detection.

### Detection With Unsupervised Learning Approaches

Three studies combined SL and UL approaches. Shen et al [6] employed four approaches of multimodal depressive dictionary learning (MDL), naive Bayesian, multiple social networking learning, Wasserstein Dictionary Learning, and they demonstrated that the proposed MDL model is effective in depression detecting, obtaining the best performance with an F1-Measure of 85%. The researchers found Twitter users’ posting behaviors contributed more to depression detection than posting content. Simultaneously, De Choudhury, Counts et al [29] adopted principal component analysis and SVM as predicting approaches, and the SVM classifier achieved a high accuracy of 73% for depression detection. The researchers pointed out that the study conducted an analysis leveraging people’s information and health behaviors, which might involve sensitive privacy and ethical issues about data protection. And the privacy and ethical issues deserved serious consideration in the process of research. Finally, the study of Mariñelarena-dondena et al [26] introduced singular value decomposition, gradient boosting machines, and synthetic minority oversampling techniques as predicting approaches, and the proposed deep learning approach performed better than other classifiers for depression detection, achieving an accuracy of over 94%.

## Discussion

### Principal Findings

Our review aimed to outline studies that conducted depression detection with ML approaches based on text from social media. According to studies included in this review, researchers would extract features from online text users posted on social media using text analysis strategies such as LIWC and other word-embedding models. Next, the researchers input the features into ML models to conduct depression prediction. The features among the seventeen studies were all produced based on words in the online text, such as emotional information, linguistic style, temporal process information, social network features, etc. As for ML approaches used in depression predicting, SL was adopted more than UL. According to the above-mentioned studies [22,23], ML approaches achieved good accuracies for depression detection using text from social media, such as Facebook, Twitter, mic-blog, etc. Nevertheless, some studies also presented that there were several challenges with ML approaches [14,15,21], and problems of piracy and popularization were ongoing concerns [18].

It is worth noting that there are some common patterns in the studies reviewed here. In terms of depression identification, the existing studies analyzed in this review are consistent in that the researchers, in each case, first identified depressed and nondepressed groups among their subjects, according to either researcher-inferred mental status, user-declared mental status, community membership, or clinicians’ judgments and then explored ways of classifying the subjects using ML approaches, and measured the accuracies of the models’ predictions. Furthermore, most of the experiments reviewed here used SL rather than UL models. UL is used to identify unobserved or underlying psychological dimensions and explore how to achieve optimal classification, while SL uses existing information in the feature database for higher-level analyses concerning identification. SL uses classifications established ahead of time to explore ways to forecast a specific outcome of interest, such as the presence of a psychiatric disorder (eg, depression and anxiety). UL explores phenomena such as clustering and compression within sets of unlabeled data [33]. Therefore, for scenarios where prediction of a specific variable, such as depression, is the aim, SL approaches may be more accurate and efficient than UL approaches [12].

Several limitations and challenges of the depression identification and predicting models reviewed here should be acknowledged. Firstly, for depression identification, the fact that some types of information about the individuals, such as sociodemographic characteristics, behaviors behind the scenes, psychological, social, and cultural environment are often lacking in social media data and pose challenges that may be hard to resolve [29,31]. Secondly, the quantities of individual users’ posts vary greatly, and posts containing too few of the terms designated as relevant input could lead to bias in depression identification [15]. Moreover, all of the study samples of the 17 studies reviewed were from either China, Japan, the United States, or other English-speaking countries. As a result of cultural and other differences, populations from different countries tend to differ in terms of posting frequencies and content, which may also lead to bias in depression identification. The generalizability of measurement standards for depression is also limited across countries and cultures [27]. It should also be mentioned that the studies reviewed here all explored ML approaches to detecting depression using only text data from social media, which may have limited their predictive efficacy. Given that social media data can also include videos, photos, etc, it may be that including more types of social media data in analyses could make depression identification programs more powerful.

The challenges facing ML approaches for depression detection may, however, be resolvable. For example, existing studies indicate that homophily exists among depressed users; that is to say, friends who interact with depressed users frequently are more likely to have depressive symptoms themselves. Therefore, the interactions and ties between users are significant. But the data used in such prediction models tend to be widely scattered on social media, and it is difficult to analyze the connections among individuals in such a way as to improve the accuracies of the ML approaches [21]. Moreover, only a large-scale data set could facilitate high accuracy in predictive applications. However, due to the characteristics of the data, it is hard to collect a sufficiently large mass of data to optimize the ML approaches applied. Often the studies are conducted based only on several hundred subjects [7,27].

In addition, the approaches and features selected for the analyses are crucial aspects of studies in this area. Wider ranges of possible features, such as specific depression lexicons appropriate for particular cultural populations or groups, and more complex techniques for analyzing posts should be explored with a view to ameliorating experimental processes and improving the accuracies of models [7]. The study conducted by De Choudhury, Gamon, et al [25], for example, in addition to using principal component analysis to perform feature reduction, also employed data abstraction techniques such as entropy, variance, and an average of the features which were significantly helpful in identifying the effects of the methods used in the study. Some approaches, however, tend to have deficiencies in both generalizability and variables selected for measurement. For example, there was a study that ended up not identifying depressed users, but only depressive tendencies, as revealed in posts on social media, because of the methods they applied [17]. Finally, there tends to be bias in the detection of depression when ML approaches are applied to data from social media. We know, for example, that youth and middle-aged people tend to be more active on social media than young children and older adults [32]. It’s also true that there is a digital divide between people with higher and lower incomes [34], and people in more developed and richer countries and localities use social media more than those in poor and undeveloped areas, etc. What’s more, most older adults seldom go on the internet. For example, according to the Pew Research Center, only 22% of American adults report using Twitter, and 73% of those people are under the age of 50 years [35]. Therefore, we cannot obtain data from social media that will represent all groups, leading to inherent population biases in studies based on social media.

To improve the validity and feasibility of depression detection research based on the application of ML approaches to social media data, increased efforts to reduce research bias will be needed. For depression identification, researchers should employ criteria and tools for depression diagnosis that are both accurate and suitable for different online populations. Moreover, collecting personal information such as sociodemographic characteristics and behaviors behind the scenes should also be considered, where necessary and ethical [31]. Furthermore, on methods used for predicting, first, it is important to refine the prediction results by continually exploring optimal input features, models, and ML approaches through constant training and learning with larger-scale samples. Second, studies should focus on standardizing the measures being used for depression detection with ML approaches and on developing scalable approaches for automated tracking of public psychological health in the future. Third, to avoid estimate biases caused by small sample sizes, researchers should focus on obtaining samples that are as large as possible for their analyses. Finally, discussions about the issues involved in the studies should include computer scientists, psychologists, clinicians, ethicists, lawyers, policymakers, as well as user representatives from various user groups.

### Conclusions

In summary, the studies described in this review have demonstrated that ML approaches can be effective for detecting depression using text data from social media and that the objective of developing a highly valid approach for such research may be within reach. Additionally, it seems appropriate and applicable for these methods to function as a complementary tool to the more traditional, established methods for diagnosing depression. However, further research is still needed in the areas of sample size, optimization of predictive approaches and features, generalizability, privacy issues, and general research ethics.

## Abbreviations

API: application programming interface
CNN: convolutional neural networks
EDDTW: event-driven depression tendency warning
LDA: latent Dirichlet allocation
LIWC: linguistic inquiry and word count
LSTM: long short term memory
MDL: multimodal depressive dictionary learning
ML: machine learning
PTT: Gossip Forum on the Professional Technology Temple
RNN: recurrent neural network
SL: supervised learning
SS3: sequential S3 (smoothness, significance, and sanction)
SVM: support vector machine
UL: unsupervised learning
WOZ-DAIC: Distress Analysis Interview Corpus-Wizard of Oz
